# Whole exome sequencing identifies *TRIOBP* pathogenic variants as a cause of post-lingual bilateral moderate-to-severe sensorineural hearing loss

**DOI:** 10.1186/s12881-017-0499-z

**Published:** 2017-12-02

**Authors:** Agnieszka Pollak, Urszula Lechowicz, Victor Abel Murcia Pieńkowski, Piotr Stawiński, Joanna Kosińska, Henryk Skarżyński, Monika Ołdak, Rafał Płoski

**Affiliations:** 1 Department of Genetics, Institute of Physiology and Pathology of Hearing, Mochnackiego 10, Warsaw, 02-042 Poland; 2 Department of Medical Genetics, Warsaw Medical University, Pawinskiego 3c, Warsaw, 02-106 Poland; 3 Postgraduate School of Molecular Medicine, Warsaw Medical University, Warsaw, Poland; 4 Oto-Rhino-Laryngology Surgery Clinic, Institute of Physiology and Pathology of Hearing, Warsaw/Kajetany, Poland

**Keywords:** Whole exome sequencing, Hearing impairment, *TRIOBP*

## Abstract

**Background:**

Implementation of whole exome sequencing has provided unique opportunity for a wide screening of causative variants in genetically heterogeneous diseases, including nonsyndromic hearing impairment. TRIOBP in the inner ear is responsible for proper structure and function of stereocilia and is necessary for sound transduction.

**Methods:**

Whole exome sequencing followed by Sanger sequencing was conducted on patients derived from Polish hearing loss family.

**Results:**

Based on whole exome analysis, we identified two *TRIOBP* pathogenic variants (c.802_805delCAGG, p.Gln268Leufs*610 and c.5014G>T, p.Gly1672*, the first of which was novel) causative of nonsyndromic, peri- to postlingual, moderate-to-severe hearing loss in three siblings from a Polish family. Typically, *TRIOBP* pathogenic variants lead to prelingual, severe-to-profound hearing loss, thus the onset and degree of hearing impairment in our patients represent a distinct phenotypic manifestation caused by *TRIOBP* variants. The pathogenic variant p.Gln268Leufs*610 disrupts the TRIOBP-4 and TRIOBP-5 isoforms (both expressed exclusively in the inner ear and retina) whereas the second pathogenic variant c.514G>T, p.Gly1672* affects only TRIOBP-5.

**Conclusions:**

The onset and degree of hearing impairment, characteristic for our patients, represent a unique phenotypic manifestation caused by *TRIOBP* pathogenic variants. Although *TRIOBP* alterations are not a frequent cause of hearing impairment, this gene should be thoroughly analyzed especially in patients with a postlingual hearing loss. A delayed onset of hearing impairment due to *TRIOBP* pathogenic variants creates a potential therapeutic window for future targeted therapies.

## Background

Two-thirds of early onset hearing impairment (HI) cases are due to genetic causes [1]. After excluding the *GJB2* and *GJB6* pathogenic variants more than 90 genes may be involved in HI pathogenesis (http://hereditaryhearingloss.org). Massive screening for causative variants within all protein-coding sequences is now available through whole exome sequencing (WES). Thus, a wide and unbiased search for pathogenic variants has become possible in many genetically heterogeneous diseases including HI [2].

*TRIOBP* (Trio- and f-actin-binding protein) (MIM 609761) was cloned in 2001 as *TARA* [3] and renamed after its mapping to chromosome 22q13.1 [4]. Multiple isoforms of the protein, differing in total length and expression pattern, have been discovered [5, 6]. Both, human and mouse isoforms are classified into long (TRIOBP-3, TRIOBP-5, TRIOBP-6) and short (TRIOBP-1, TRIOBP-2, TRIOBP-4). Interestingly, no part of the protein is shared between TRIOBP-1 and TRIOBP-4 [3]. Such a variety of isoforms derived from a single gene may be explained by the presence of six putative alternative promoters [7].

While TRIOBP-1 is widely expressed in different tissues, TRIOBP-4 and TRIOBP-5 was exclusively found in the adult inner ear and retina of both human and mice. In the inner ear TRIOBP-4 and TRIOBP-5 are expressed in stereocilia rootlets. Additionally, TRIOBP-4 is also localized along the whole length of stereocilia. Proper structure of the rootlets is essential for stereocilia rigidity and stiffness, thereby allowing normal process of sound transmission [8]. In contrast, TRIOBP-1 plays a role in regulation of adherens junctions as well as reorganization of the actin cytoskeleton, especially in stress fibers and cortical F-actin [6].

We present here a family with isolated, perilingual to postlingual hearing loss with a recessive inheritance pattern, in which WES followed by direct Sanger sequencing allowed to identify one novel (c.802_805delCAGG, p.Gln268Leufs*610) and one known (c.5014G>T, Gly1672* [9]) *TRIOBP* pathogenic variant and establish a molecular diagnosis.

## Methods

**Fig. 1:**
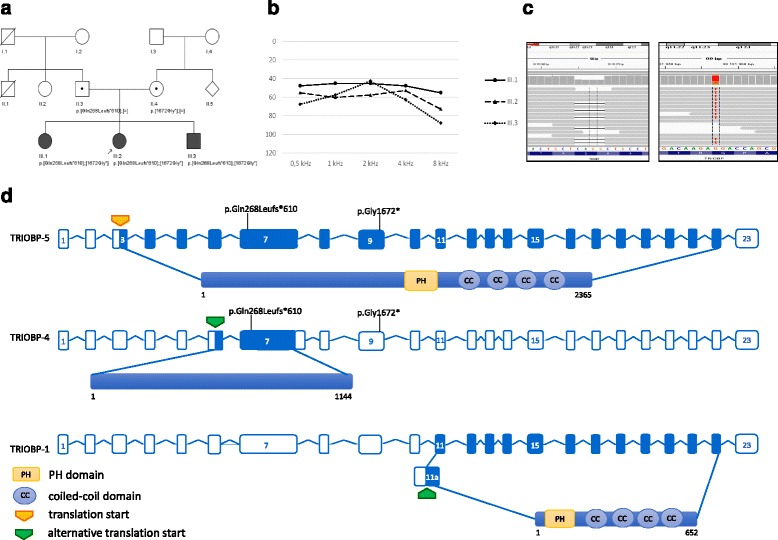
Structure and variants of *TRIOBP* in the studied family with HI. **a**. Pedigree of the analyzed family. Circles and squares represent females and males, respectively. Black arrow denotes the proband, genotypes of the *TRIOBP* gene are given below the respective symbols. **b**. Mean audiograms for both ears in all affected siblings (III.1, III.2, III.3) (Y axis presents HI level in dB, X axis presents the tested frequency in kilohertz). **c**. *TRIOBP* pathogenic variants identified in this study - Integrative Genomics Viewer (IGV) views for c.802_805delCAGG, p.Gln268Leufs*610 (left panel) and c.5014G>T, p.Gly1672* (right panel). **d**. Schematic localization of the c.802_805delCAGG, p.Gln268Leufs*610 and c.5014G>T, p.Gly1672* variants within the *TRIOBP* gene. Upper panels show the *TRIOBP* gene structure, whereas the lower panels present three isoforms, TRIOBP-5, TRIOBP-4 and TRIOBP-1, respectively. All of them are in the same reading frame. TRIOBP-4 translation starts from an alternative start site in exon 6, whereas TRIOBP-1 translation start is localized within an alternative exon 11a. Numbers given below the particular isoforms denote first and last amino acid

Pedigree of the analyzed family is presented in Fig. 1a. The level of hearing loss was determined based on pure tone audiometry (PTA) and classified as perilingual if its onset was between 3 to 6 y or postlingual with an onset after 6 y.

DNA was isolated from peripheral blood by a standard salting out method [10]. In the affected siblings *GJB2* pathogenic variants and *GJB6* deletions were excluded [11]. Library for WES was prepared with TruSeq Exome Enrichment Kit according to the manufacturer’s protocol (Illumina Inc., San Diego, CA, USA), subsequently the sample was run on HiSeq 1500 (Illumina Inc.) with 2 × 100 bp paired-end reads. After obtaining the coverage of minimum 20 times on at least 80% of the target, bioinformatics analysis was performed as previously described [12]. All retrieved variants were annotated with Annovar and presented in MS Access database for further expert investigations. The DNA sequences were viewed with Integrative Genomic Viewer (IGV) [13, 14].

Potentially pathogenic variants in *TRIOBP* were confirmed by Sanger sequencing with the use of dedicated primers (sequences available upon request) designed with the Primer 3 software [15] based on the NM_001039141.2 reference sequence.

The prediction of presence of nonsense-mediated mRNA decay (NMD) mechanism was performed with the Mutation Taster algorithm [16].

Exome Aggregation Consortium (ExAC) (http://exac.broadinstitute.org) (accessed 12/2016) and the in-house database of WES data from the Polish population (*n* = 1052) were used for the assessment of the population prevalence of the detected variants.

## Results

### Phenotypic manifestation

Proband (III.2), as well as the two affected siblings suffered from bilateral, non-syndromic HI. Apart from HI, all of the siblings were otherwise healthy. The age of HI onset was 12 y for the proband and 4,5 y and 3 y for the sibling III.1 and III.3, respectively. The proband was reported for an audiology consultation due to suspected hearing loss based on the parents’ observation that she listened to electronic devices on a loud volume. The reason for the audiology tests in the siblings was similar, but it cannot be excluded that the parents were more attentive in observing their younger son. All siblings suffered from moderate-to-severe HI, detailed audiometric data are presented in Fig. 1b. The 2-year follow-up does not show hearing deterioration in sibling III.1 and III.2, for the patient III.3 the additional audiometric data were not available. Notably, the youngest brother (III.3) passed the newborn hearing screening test, the remaining sibs did not have this test. Patient III.3 suffers from frequent otitis media and he has a relevant air-bone gap in the right ear (~40 dB). Now his HI is classified as mixed comprising of a conductive and sensorineural component.

### Variants detection

WES performed in patient III.2 revealed the presence of two, potentially pathogenic variants in the *TRIOBP* gene (NM_001039141.2:c.802_805delCAGG, NP_001034230.1: p.Gln268Leufs*610; NM_001039141.2:c.5014G>T, NP_001034230.1:p.Gly1672*) both in a heterozygous form (Fig. 1c). The variant c.802_805delCAGG, p.Gln268Leufs*610 was novel, whereas c.5014G>T, NP_001034230.1:p.Gly1672* was already known [9]. Direct Sanger sequencing of the region encompassing the detected variants confirmed the presence of both variants in the proband and her affected siblings. Analysis of parents’ DNA showed that they were both heterozygous carriers, thus establishing an in trans configuration of the variants in the affected children (Fig. 1a). The c.802_805delCAGG, p.Gln268Leufs*610 mutation occurs with an allele frequency of 0,000008 and 0,00095 in ExAC and the Polish in-house WES databases, respectively. Allele frequency for c.5014G>T, p.Gly1672*, the second identified *TRIOBP* variant, was 0,0006 and 0,002 in the same databases (Table 1).

**Table 1:** Mutations in the *TRIOBP* gene (NM_001039141.2, NP_001034230.1) according to The Human Gene Mutation Database (HGMD), their frequency in ExAC and Polish in-house WES database and available clinical data

variant	phenotype	ExAC allele count	ExAC allele frequency	Pol_Exome allele count	Pol_Exome allele frequency	reference	age of onset	HI level	ethnicity	pathogenicity of variant^d^
c.154G>A (p.Asp52Asn)	deafness	23/117210	0,0001962	0	0	[ 17 ]	late onset^a^	nd	Japanese	likely pathogenic (PM2)
**c.802_805delCAGG (p.Gln268fs)**	postlingual hearing loss	1/120722	0,000008283	2	0,0019	this study	postlingual	moderate-to-severe	Polish	pathogenic (PVS1)
c.889C>T (p.Gln297^*a^)	deafness non-syndromic	0^c^	0^c^	0	0	[ 5 ]	prelingual	severe-to-profound	Indian	pathogenic (PVS1)
c.1039C>T (p.Arg347^*a^)	deafness non-syndromic	5/120762	0,0000414	0	0	[ 6 ]	prelingual	profound	Palestinian	pathogenic (PVS1)
c.1741C>T (p.Gln581^*a^)	deafness non-syndromic	0^c^	0^c^	0	0	[ 6 ]	prelingual	profound	Palestinian	pathogenic (PVS1)
c.2355_2356delAG (p.Arg785fs)	Hearing loss non-syndromic autosomal recessive	0^c^	0^c^	0	0	[ 2 ]	nd	nd	Turkish	pathogenic (PVS1)
c.2362C>T (p.Arg788^*a^)	deafness non-syndromic	4/120766	0,00003312	0	0	[ 5 ]	prelingual	severe-to-profound	Pakistani	pathogenic (PVS1)
c.2581C>T (p.Arg861^*a^)	hearing loss non-syndromic autosomal recessive	1/120758	0,000008281	0	0	[ 31 ]	congenital	profound	Chinese	pathogenic (PVS1)
c.2653del (p.Arg885fs)	hearing loss	0^c^	0^c^	0	0	[ 9 ]	prelingual	moderate	Dutch	pathogenic (PVS1)
c.2758C>T (p.Arg920^*a^)	Hearing loss non-syndromic autosomal recessive	0^c^	0^c^	0	0	[ 31 ]	congenital	profound	Chinese	pathogenic (PVS1)
c.2992G>A (p.Ala998Thr)	hearing loss	85/120672	0,0007044	0	0	[ 18 ]	congenital	mild to moderate	unknown	likely pathogenic (PM2)
c.3055G>A (p.Gly1019Arg)	deafness non-syndromic	0^c^	0^c^	0	0	[ 6 ]	prelingual	profound	Palestinian	likely pathogenic (PM2)
c.3202_3203delCG (p.Asp1069fs)	deafness non-syndromic	0^c^	0^c^	0	0	[ 5 ]	prelingual	severe-to-profound	Indian	pathogenic (PVS1)
c.3202C>T (p.Arg1068^*a^)	deafness non-syndromic	1/120342	0,00000831	0	0	[ 5 ]	prelingual	severe-to-profound	Pakistani	pathogenic (PVS1)
c.3232dupC (p.Arg1078fs)	deafness non-syndromic	0^c^	0^c^	0	0	[ 5 ]	prelingual	severe-to-profound	Indian	pathogenic (PVS1)
c.3232C>T (p.Arg1078Cys)	hearing loss non-syndromic autosomal recessive	355/120198	0,002953	0	0	[ 32 ]	prelingual	nd	Western-European	likely pathogenic (PM2)
c.3349C>T (p.Arg1117*^a^)	deafness non-syndromic	0^c^	0^c^	0	0	[ 5 ]	prelingual	severe-to-profound	Indian	pathogenic (PVS1)
c.3451A>G (p.Met1151Val)	hearing loss non-syndromic autosomal recessive	14/120634	0,0001161	0	0	[ 31 ]	nd	severe to profound HL of the left ear, and mild to moderate HL of the right ear	Chinese	uncertain significance^f^
c.3460_3461del (p.Leu1154fs)	hearing loss	5/120614	0,00004145	0	0	[ 9 ]	prelingual	mild	Dutch	pathogenic (PVS1)
c.3662G>A (p.Arg1221Gln)	hearing loss	38/116270	0,0003268	0	0	[ 18 ]	congenital	severe to profound	unknown	uncertain significance^e^
c.3942G > C (p.Glu1314Asp)	hearing loss	3/7416	0,0004045	0	0	[ 18 ]	congenital	severe to profound	unknown	uncertain significance^e^
c.4187C>T (p.Pro1396Arg)	hearing loss non-syndromic autosomal recessive	1/69904	0,00001431	0	0	[ 31 ]	nd	severe to profound HL of the left ear, and mild to moderate HL of the right ear	Chinese	uncertain significance^f^
c.4691G>C (p.Gly1564Ala)	hearing loss	0^c^	0^c^	0	0	[ 18 ]	nd	Nd	unknown	likely pathogenic (PM2)
c.4840G>T (p.Gly1614Cys)	deafness	18/118002	0,0001525	0	0	[ 17 ]	early onset^b^	Nd	Japanese	likely pathogenic (PM2)
c. 5014G>T (p.Gly1672*^a^)	postlingual hearing loss	59/103272	0,0005713	4	0,0038	this study/ [9]	postlingual/prelingual	moderate-to-severe/moderate	Polish/Dutch	pathogenic (PVS1)
c.5519G>A (p.Arg1840His)	deafness	3/113104	0,00002652	0	0	[ 17 ]	early onset^b^	nd	Japanese	likely pathogenic (PM2)
c.5767G>A (p.Ala1923Thr)	hearing loss	47/31044	0,001514	0	0	[ 18 ]	congenital	mild to moderate	unknown	likely pathogenic (PM2)
c.6362C>T (p.Ser2121Leu)	hearing loss non-syndromic autosomal recessive	56/19236	0,002911	0	0	[ 25 ]	prelingual	nd	Iranian	likely pathogenic (PM2)
c.6736G>A (p.Glu2246Lys)	hearing loss	556/90848	0,00612	0	0	[ 18 ]	congenital	severe to profound	unknown	likely pathogenic (PM2)
c.6860G>A (p.Arg2287His)	deafness	0^c^	0^c^	0	0	[ 17 ]	early onset^b^	nd	Japanese	likely pathogenic (PM2)
c.7000C>T (p.Arg2334Trp)	hearing loss non-syndromic autosomal recessive	2/120504	0,0000166	0	0	[ 32 ]	prelingual	nd	Western-European	likely pathogenic (PM2)

Novel mutation is written in bold

*nd* no data

^a^ above 6 y.o

^b^ below 6 y.o

^c^not reported in ExAC database

^d^according to criteria for classifying pathogenic variants described by Richards et al. [26]; PVS1 - very strong evidence of pathogenicity (null varaints in a gene where loss of function is a known mechanism of disease); PM2 moderate evidence of pathogenicity (absent from controls or at low frequency in ExAC)

^e^found in cis with second TRIOBP variant

^f^ in silico predictions are contradictory

The c.802_805delCAGG, p.Gln268Leufs*610 variant (localized within exon 7) introduces a 4-nucleotide deletion, which results in a frameshift, leading to shortening of the protein after the following 610 amino acids, while the c.5014G>T, p.Gly1672* variant (localized within exon 9) directly introduces a stop codon. Presence of two deleterious *TRIOBP* variants in a trans configuration in all affected children strongly suggest that they represent a molecular cause of HI in these patients.

## Discussion

Here we report on the first *TRIOBP* family suffering from perilingual or postlingual, moderate-to-severe HI. At the time of writing the onset and degree of HI, characteristic for our patients, represent a unique phenotypic manifestation caused by *TRIOBP* pathogenic variants. Most of the heretofore detected *TRIOBP* pathogenic variants are related to prelingual, severe-to-profound hearing loss. There is only one variant (c.154G>A, p.Asp52Asn) assigned to postlingual onset of HI [17] but in this patient the level of HI has not been given. On the other hand, there are few reports on a less severe HI in patients with *TRIOBP* variants but with a prelingual onset [18] (Table 1). Thus, our *TRIOBP* family is the first one with a less severe HI in respect to both its onset and level.

To date, little is known about the exact function of TRIOBP. It contains two types of relevant domains, i.e. N-terminal Pleckstrin Homology (PH) and C-terminal coiled-coil. It is established that this protein directly binds and stabilizes the F-actin structures [3], presumably via nonconventional actin-binding sites localized in the coiled-coil domains or PH domains [19, 20]. As its name suggests, Triobp interacts with Trio, a protein derived from a group of Dbl-homology guanine nucleotide exchange factors (DH-GEFs) [3]. DH-GEFs family of proteins controls reorganization of actin cytoskeleton, cell adhesion, and also serves as transcription factors due to activation of Rho GTPases [21]. Multiple roles of TRIOBP, such as its involvement in organization of actin-cytoskeleton [3], proper centrosomal localization and segregation of chromosomes during cell division [22], cell cycle regulation in conjunction with HECTD3 protein [23] or regulation of cancer cells motility [24] raise the issue why pathogenic variants in this gene do not lead to other pathologies than isolated hearing loss. In the family presented here, both detected variants affects TRIOBP-5, whereas c.802_805deCAGG, p.Gln268Leufs*610 affects also TRIOBP-4, without impairing the most widely expressed TRIOBP-1 (Fig. 1d), which may explain the selectivity of symptoms. On the other hand, recently some likely pathogenic variants affecting also TRIOBP-1 were described (e.g. homozygous c.6362C>T, p.Ser2121Leu [25]), leading to bilateral, prelingual HI with no other symptoms, thus the comprehensive explanation of this phenotypic phenomena needs further study.

Both variants detected in this study are predicted to result in a premature termination of translation (after amino acid 878 and 1672, respectively). Shortened TRIOPB protein due to c.802_805deCAGG, p.Gln268Leufs*610 and c.5014G>T, p.Gly1672* variants is devoid of PH and coiled-coil domains crucial for the actin-binding process. There is also a possibility that the impaired TRIOBP transcripts are directed to degradation via the NMD mechanism, which was suggested by the Mutation Taster algorithm [16]. The conclusive verification of this prediction needs further study; however, due to a restricted expression of TRIOBP-5 and TRIOBP-4 only to the inner ear and the retina we could not test the processing of TRIOBP transcripts in our patients. According to the Criteria for Classifying Pathogenic Variants described by Richards et al. [26], both variants have been classified into a group of very strong evidence of pathogenicity (PVS1) based on their type (nonsense and frameshift), localization within a gene where loss of function is a known mechanism of HI, and low allelic frequency in the ExAC and Polish in-house databases. Additionally, a paper recently published by Wesdorp et al. [9] confirms the pathogenicity of the c.5014G>T, p.Gly1672* variant. Taking into account all the above and the fact that both variants were found in all three affected siblings, we consider c.802_805deCAGG, p.Gln268Leufs*610 and c.5014G>T, p.Gly1672* as causative for HI.

A putative consequence of the described variants is a faulty or absent (via NMD) TRIOBP protein, presumably incapable to form stereocilia rootlets. In response to sound stimulation stereocilia dispossessed of their rootlets, deflect two-four times more intensely than the proper ones and are vulnerable to damage due to their floppiness [27]. These features are consistent with the phenotype observed in the studied family – perilingual or postlingual, moderate-to-severe hearing loss. Furthermore, the localization of the c.5014G>T, p.Gly1672* affecting only the TRIOBP-5 isoform let us assume that the remaining dose of the TRIOBP-4 protein may allow an impaired functioning of the stereocilia, thus leading to a less severe phenotype than those when both isoforms are affected. This is in line with the results of Kitajiri et al. showing that TRIOBP-4 alone is sufficient to form condensed bundles from filamentous-actin parallel to stereocilia rootlets [8]. Similar, milder phenotype (moderate HI) was reported by Wesdorp et al. [9] in a family harboring pathogenic variants: c.2653del, p.Arg885fs*120 and c.5014G>T, p.Gly1672*, although in this case the HI was congenital. It is worth noting that one of the here described siblings passed the newborn screening test (unfortunately the other affected siblings were not tested), which strongly supports that the trigger mechanism damaging hair cells occurs after birth. Considering the role of stereocilia rootlets in adaptation to mechanical stress due to acoustic trauma [27] it is possible that patients harboring truncating variants in the *TRIOBP* gene are more prone to acoustic trauma. Thus, it seems essential for *TRIOBP* patients to rigorously avoid noise. On the other hand, a confirmed postnatal origin of HI provides a therapeutic window for targeted treatment in the future.

*TRIOBP* variants are not a frequent cause of HI. To date, 31 point alterations were described (Table 1) in the *TRIOBP* gene, with HI being the only phenotypic manifestation. Most of heretofore described pathogenic or likely pathogenic variants are located within exon 7, thus this region should be considered as hotspot. Accumulation of repetitive sequences within this area of *TRIOBP*, may render it more prone to mutation than others [28]. Taking into account the frequency of mutated *TRIOBP* alleles in the ExAC database, c.5014G > T, p.Gly1672* is the fourth most common pathogenic variant in this gene, additionally this variant is the most prevalent in the Polish population (Table 1). The data highlights the population distinctiveness and necessity of a preliminary population analysis before introducing a diagnostic test. Thus, in a small diagnostic gene panel for HI at least the regions encompassing common *TRIOBP* variants (c.6736G>A, p.Glu2246Lys; c.3232dupC, p.Arg1078Cys; c.2992G>A, p.Ala998Thr and c.5014G>T, p.Gly1672*) should be included. Considering their low frequency in ExAC and our Polish in-house WES databases, point variants in the *TRIOBP* gene seem to be a rare but important cause of non-syndromic hearing loss. Given the high heterogeneity of the HI [29], among patients with unknown cause of this disorder, after exclusion of the *GJB2* and *GJB6* pathogenic variants, wide screening for causative mutations using next generation sequencing is becoming a standard with at least 18 targeted diagnostic HI panels described to date [30].

## Conclusions

Here we report one novel and one known *TRIOBP* pathogenic variant (c.802_805delCAGG, p.Gln268fs and c.5014G>T, p.Gly1672*) causative of a unique phenotype. The presented variants disrupt isoform: TRIOBP-4, TRIOBP-5 and TRIOBP-5 respectively, which are expressed in the inner ear and form stereocilia rootlets. Although *TRIOBP* variants are not a frequent cause of hearing loss, this gene should be thoroughly analyzed also in patients with postlingual HI.

## Abbreviations

DH-GEFs: Dbl-homology guanine nucleotide exchange factors
ExAC: Exome Aggregation Consortium
HGMD: The Human Gene Mutation Database
HI: Hearing impairment
PH: N-terminal Pleckstrin Homology
PTA: Pure tone audiometry
TRIOBP: Trio- and f-actin-binding protein
WES: Whole exome sequencing
